# Oxidation Damage Evolution in Low-Cycle Fatigue Life of Niobium-Stabilized Austenitic Stainless Steel

**DOI:** 10.3390/ma15124073

**Published:** 2022-06-08

**Authors:** Wan-Kyu Choi, Sangyul Ha, Jong-Cheon Kim, Jong-Cheon Park, Aokai Gong, Tae-Won Kim

**Affiliations:** 1 Department of Automotive Engineering, Hanyang University, Seoul 04763, Korea; wanguu@hanyang.ac.kr; 2 Department of Mechanical Engineering, Gachon University, Seongnam-si 13306, Korea; dubuking@postech.ac.kr; 3 Research & Development Headquarter, Hyundai Motor Company, Hwaseong-si 18280, Korea; kjc8363@hyundai.com (J.-C.K.); skybell@hyundai.com (J.-C.P.); 4 Department of Mechanical Engineering, Hanyang University, Seoul 04763, Korea; gak971230@hotmail.com

**Keywords:** austenitic stainless steel, chromium carbide, oxidation damage, fatigue life, low-cycle fatigue

## Abstract

Austenitic stainless steel is a vital material in various industries, with excellent heat and corrosion resistance, and is widely used in high-temperature environments as a component for internal combustion engines of transportation vehicles or power plant piping. These components or structures are required to be durable against severe load conditions and oxidation damage in high-temperature environments during their service life. In this regard, in particular, oxidation damage and fatigue life are very important influencing factors, while existing studies have focused on materials and fracture behavior. In order to ensure the fatigue life of austenitic stainless steel, therefore, it is necessary to understand the characteristics of the fracture process with microstructural change including oxidation damage according to the temperature condition. In this work, low-cycle fatigue tests were performed at various temperatures to determine the oxidation damage together with the fatigue life of austenitic stainless steel containing niobium. The characteristics of oxidation damage were analyzed through microstructure observations including scanning electron microscope, energy-dispersive X-ray spectroscopy, and the X-ray diffraction patterns. In addition, a unified low-cycle fatigue life model coupled with the fracture mechanism-based lifetime and the Neu-Sehitoglu model for considering the influence of damage by oxidation was proposed. After the low-cycle fatigue tests at temperatures of 200–800 °C and strain amplitudes of 0.4% and 0.5%, the accuracy of the proposed model was verified by comparing the test results with the predicted fatigue life, and the validity by using the oxidation damage parameters for Mar-M247 was confirmed through sensitivity analysis of the parameters applied in the oxidation damage model. As a result, the average thickness of the oxide layer and the penetration length of the oxide intrusion were predicted with a mean error range of 14.7% and 13%, respectively, and the low-cycle fatigue life was predicted with a ±2 factor accuracy at the measurement temperatures under all experimental conditions.

## 1. Introduction

Austenitic stainless steel is an important material in many industries, including automotive engines, exhaust systems, aircraft gas turbines, home appliance motors, and power plant turbine rotors, and can maintain durability with its heat and corrosion resistance, especially in high-temperature environments of 600 °C or higher.

However, when austenitic stainless steel is used in the sensitization temperature range, i.e., 400–850 °C, the affinity of chromium (Cr) and carbon (C) increases, and chromium carbide (Cr_23_C_6_), a type of metal carbide (M_23_C_6_), is precipitated inside the material, and the amount of chromium for suppressing the oxidation of the material decreases as carbon and chromium bond with each other for the precipitation of Cr_23_C_6_ [1,2,3]. During this process, the thin chromium oxide layer (Cr_23_O_3_), which exists on the surface of the austenitic stainless steel and serves as a protective film from the external environment, is depleted, and an oxide (Fe_x_O_y_) is formed on the surface of the material [4,5]. When a mechanical load is subsequently applied to the material, the oxide is damaged, and the metal inside the material is exposed to the external environment through open cracks. As a result, damage occurs as the newly formed oxide penetrates the material, and this oxidation damage process is repeated when a fatigue load is applied to the material at a high temperature, affecting the mechanical properties and fatigue life of the material [6].

In order to reduce this oxidation damage in the sensitization range, niobium is added as a stabilizing element to austenitic stainless steel to suppress Cr_23_C_6_ precipitation and prevent oxide formation [5,7]. While heat treatment can be performed for stabilization at a high temperature of 950 °C or higher to make it difficult for Cr_23_C_6_ to form and adequately maintain the effect of adding niobium [8], various unexpected deformations may occur in the material [9]. Therefore, considering these issues, there is a need for research on the oxidation, oxidation damage, and the resulting fatigue life of austenitic stainless steel containing niobium that is not heat-treated for stabilization.

Meanwhile, the failure of material in a periodic-load environment at high temperatures can not only lead to damage by fatigue but also damage by oxidation mechanism, thus requiring a model that can reflect both of these damage mechanisms for a more precise prediction of the fatigue life of austenitic stainless steel [10]. The Coffin-Manson or Smith-Watson-Toper models have been used to predict the low-cycle fatigue (LCF) life but are limited in predicting accurate fatigue life by reflecting the damage mechanisms, such as fatigue and oxidation in a high-temperature region [11,12,13]. Sehitoglu et al. proposed the Neu-Sehitoglu model that classified fatigue damages occurring in a high-temperature, periodic-load environment into fatigue, creep, and oxidation damages [14,15,16]. Karl applied this Neu-Sehitoglu model to predict the life of austenitic stainless steel under isothermal LCF load conditions [17], and Wang used the Neu-Sehitoglu model to predict the isothermal LCF life of exhaust manifolds made of stainless steel based on fatigue and oxidation damages [18]. As such, the Neu-Sehitoglu model can be used to predict the fatigue life through linear superposition after respectively calculating fatigue, creep, and oxidation damages; however, these approaches by using the Neu-Sehitoglu could not reflect the detailed mechanisms associated with the growth of LCF cracks, and consequentially the effects of time, temperature, strain amplitude, and mean stress on crack growth.

Since microcracks are nucleated early in the life stage, the growth of microcracks under periodic loads is a life-limited damage mechanism and the growth of cracks caused by a fatigue load on the material can be evaluated by means of the fracture mechanics [19,20,21]. Schmitt et al. proposed a fracture mechanism-based lifetime model to predict fatigue life by considering the correlation between the growth of cracks and crack-tip opening displacement [22]. Although the model was originally developed to predict the fatigue life for thermo-mechanical fatigue (TMF) that simultaneously controlled the temperature and mechanical strain, the lifetime in an isothermal LCF test at a constant temperature ($\dot{T}=0$) over time could also be predicted with high accuracy [23]. Herein, crack-tip opening displacement was defined through the cyclic J-integral based damage variable only, and hence the lifetime could be predicted by considering the fatigue crack growth [24].

Previous studies on predicting the fatigue life of austenitic stainless steel usually consider the crack growth caused by fatigue load while giving less consideration to the oxidation characteristics of the material [25,26,27]. Otherwise, only the effect of oxidation damage on fatigue life was analyzed experimentally and not reflected in the prediction model [28,29]. However, in order to predict the fatigue life of austenitic stainless steel in a high-temperature environment, both the oxidation damage of the material affecting the growth rate of the crack and the effect of the characteristics of the crack tip under the fatigue load should be considered. Also, since the austenitic stainless steel has different oxidation damage as its microstructure changes depending on temperature conditions, it is necessary to understand the effect of material characteristics on fatigue life.

In this study, isothermal LCF tests were conducted on stainless steel containing niobium that was not heat-treated for stabilization, and the effects of an increased temperature and strain amplitude on the oxidation behavior of the material were observed. The suitability of the Neu-Sehitoglu oxidation damage model was investigated by predicting the stratified thickness of the oxide layer and the penetration length of oxide intrusion after the LCF tests, and the fatigue life was predicted by proposing a unified low-cycle fatigue life model coupled with the fracture mechanism-based lifetime model and the Neu-Sehitoglu oxidation damage model. Lastly, sensitivity analysis was performed on the parameters used for oxidation damage to verify the validity of the lifetime prediction methodology.

## 2. Fatigue Life Prediction Models

Based on the isothermal LCF experiment results, austenitic stainless steel containing niobium tested at a high temperature may exhibit both fatigue and oxidation damages [30,31,32], and a model needs to reflect such damage mechanisms to predict the lifetime. A unified LCF life prediction model considering the phenomena is proposed such as the fracture mechanism-based lifetime related to the crack-tip blunting model and the Sehitoglu model to evaluate the fatigue life by oxidation in a high-temperature environment. As shown in Equation (1), the model can be expressed by the reciprocal summation since the damage is equal to 1/N, assuming linear damage and also is equal to 1 at failure. In here, N is fatigue life, and subscripts, f, fatigue, and env indicate final, fatigue, and oxidation respectively.

(1) $$\frac{1}{N_{f}}=\frac{1}{N_{fatigue}}+\frac{1}{N_{env}}$$

### 2.1. Fracture Mechanism-Based Lifetime Model

If fatigue load is applied to the austenitic stainless steel containing niobium, the dislocation pile-ups occur at the obstacle and plastic deformation occurs in this area by the fatigue load. A cavity is formed here and grows into a microcrack [33,34]. The crack grows under repeated fatigue loads, which is associated with blunting in the loading cycle followed by re-sharpening of the crack–tip during unloading [35]. And crack reaches the critical crack length, causing fatigue fracture. Nucleation of microcracks is usually observed in the first few percent of life in metallic materials under LCF conditions resulting in the growth of the microcracks into critical sizes or fractures. The fatigue crack growth in ductile materials is that crack-tip blunting exposes fresh surface under the tensile load, and the surface is quickly covered by oxygen so that it cannot be re-welded at the compressive load [36,37]. Herein, based on the crack-tip blunting model, it is assumed that the crack propagation per loading cycle, da/dN, is correlated to the crack-tip opening displacement in the growth of microcracks, which is dependent on time and temperature in a fatigue test [24,38].

(2) $$\frac{da}{dN}=\beta \cdot \Delta CTOD^{B}$$

The proportional constant, β, and the index, B, are material properties, and the crack-tip opening displacement (CTOD) can be expressed as follows based on the analytically estimated cyclic J-integral, Z.

(3) $$\Delta CTOD=d_{n^{\prime }}\cdot \frac{Z}{\sigma_{CY}}$$

where $\sigma_{CY}$ is the cyclic yield stress obtained by 0.2%-offset from the stress-strain hysteresis loop, and $d_{n^{\prime }}$ is a function of the Ramberg-Osgood hardening exponent, $n^{\prime }$, given by Shih for third order polynomial function [39]:

(4) $$d_{n^{\prime }}=0.78627-4.41692n^{\prime }+6.11945{n^{\prime }}^{2}-4.2227{n^{\prime }}^{3}$$

The cyclic J-integral, Z can be approximated by the additive decomposed into elastic and plastic contribution

(5) $$Z=\left(1.45\frac{{\left(\Delta \sigma_{eff}\right)}^{2}}{E}+\frac{2.4}{\sqrt{1+3n^{\prime }}}\Delta \sigma \Delta \varepsilon^{p}\right)a=Z_{d}a$$

The damage parameter $Z_{d}$ is a parameter from the J integral of the cyclic hysteresis loop, as proposed by Heitmann, to simulate the fatigue failure behavior caused by the propagation of microcracks [40]. Here E is Young’s modulus, Δσ, $\Delta \varepsilon^{p}$ and a are the stress, plastic strain range, and crack length respectively, and the subscript, eff, indicates that the mean stress effect of a crack closure is reflected by the Newman’s crack opening stress equation [41].

Therefore, the effective stress range, $\Delta \sigma_{eff}$, and the crack opening stress, $\sigma_{op}$, are determined by the Newman equation as shown in Equation (6)

(6) $$\Delta \sigma_{eff}=\frac{\left(1-\sigma_{op}/\sigma_{max}\right)}{1-R}\Delta \sigma$$

and $\sigma_{max}$ and R are the maximum stress and the ratio of the minimum and maximum stress, respectively. Then, by the integration of Equation (2) from initial crack length $a_{o}$ to the crack length at fracture $a_{f}$, the fatigue life $N_{fatigue}$ can be calculated by

(7) $$N_{fatigue}=A\cdot {\left(d_{n^{\prime }}\cdot \frac{Z_{d}}{\sigma_{CY}}\right)}^{-B}\;with\;A=\frac{1}{\beta }\cdot \left(\frac{a_{f}^{1-B}-a_{0}^{1-B}}{1-B}\right),$$

where *A*, is a function of the initial crack length, fracture crack length, and the proportional constant, but the fatigue life calculated in the lifetime prediction model can be compared to the fatigue life measured in the LCF test to obtain the fitting parameter, *A* and *B*. Based on the LCF test results in this study, appropriate parameters were determined for the mechanism-based lifetime model of austenitic stainless steel containing niobium to predict the lifetime within a factor of 2.

### 2.2. Oxidation Damage Model

Damage that is caused by oxidation starts with crack nucleation of surface oxides and includes crack growth. Oxidation damage is evolved by crack nucleation from the rupture of the oxide layer formed on the surface, and the crack growth induced by the oxide is caused by the repeated formation and breakage of the oxide layer at the crack tip [14]. In general, the growth of oxide on the surface of a stainless steel material when an oxidation crack develops follows the parabolic growth law. Based on this, Sehitoglu et al. simulated the oxide growth in the presence of a chromium-depleted layer in the material, as shown in Equation (8) [14,15].

(8) $$h_{0}=\sqrt{(K_{p}^{ox}+K_{p}^{\gamma^{\prime }})t}$$

where, $h_{0}$ is the overall penetration length of oxide intrusion, t is the exposure time of the metal surface to the environment, and $K_{p}^{ox}$ and $K_{p}^{\gamma }$ are the parabolic oxidation constant and the parabolic $\gamma^{\prime }$ depleted layer constant, respectively, as expressed in Equations (9) and (10).

(9) $$K_{p}^{ox}=D_{ox}\exp\left(-\frac{Q_{ox}}{RT}\right)$$

(10) $$K_{p}^{\gamma^{\prime }}=D_{\gamma^{\prime }}\exp\left(-\frac{Q_{\gamma^{\prime }}}{RT}\right),$$

where, $D_{ox}$ and $D_{\gamma^{\prime }}$ are the oxidation diffusion coefficient and $\gamma^{\prime }$ depleted layer diffusion coefficient, respectively, $Q_{ox}$ and $Q_{\gamma^{\prime }}$ are the oxidation activation energy and $\gamma^{\prime }$ depleted layer activation energy, respectively, R is the gas constant, and T is the absolute temperature. Based on these equations, the oxide growth on the material surface can be predicted.

Furthermore, Sehitoglu et al. defined the propagation of the newly formed oxide penetrating inside the material through an open crack when the oxide was damaged by an external load, as shown in Equation (11).

(11) $$h_{0}=B\frac{\left(K_{peff}^{ox}+K_{peff}^{\gamma^{\prime }}\right)}{\bar{h_{f}}}t^{\beta }$$

Here, B is the time to reach the critical oxide layer thickness, β is the oxidation crack rate index, $K_{peff}^{ox}$ and $K_{peff}^{\gamma^{\prime }}$ are the effective hyperbolic oxidation coefficient and effective hyperbolic $\gamma^{\prime }$ depleted layer coefficient, respectively, which can be expressed as shown in Equations (12) and (13).

(12) $$K_{peff}^{ox}=\frac{1}{t_{c}}\int_{0}^{t_{c}}\left[D_{ox}\exp\left(-\frac{Q_{ox}}{RT\left(t\right)}\right)\right]dt$$

(13) $$K_{peff}^{\gamma^{\prime }}=\frac{1}{t_{c}}\int_{0}^{t_{c}}\left[D_{\gamma^{\prime }}\exp\left(-\frac{Q_{\gamma^{\prime }}}{RT\left(t\right)}\right)\right]dt$$

where $t_{c}$ is the duration of one cycle, that is, $2\Delta \varepsilon_{mech}/{\dot{\varepsilon }}_{mech}$, $\Delta \varepsilon_{mech}$ is the mechanical strain range, and $\dot{\varepsilon }$ is the strain rate. In addition, T(t) represents the absolute temperature function over time, and $\bar{h_{f}}$ is the average oxide layer thickness in oxide intrusion when oxidation causes a crack, as defined in Equation (14).

(14) $$\bar{h_{f}}=\frac{\delta_{0}}{{\left(\Delta \varepsilon_{mech}\right)}^{2}\Phi^{ox}{\dot{\varepsilon }}^{\alpha }}$$

Here, $\delta_{0}$ is the degree of ductility of the oxide, α is the strain rate sensitivity, and $\Phi^{ox}$ is the phase factor, which is the value obtained by dividing the integration of the phase function, $\phi^{ox}$, with respect to time by the cycle time, as shown in Equation (15)

(15) $$\Phi^{ox}=\frac{1}{t_{c}}\int_{0}^{t_{c}}\phi^{ox}dt$$

(16) $$\phi^{ox}=\exp\left[-\frac{1}{2}{\left(\frac{\dot{\varepsilon_{th}}/\dot{\varepsilon_{mech}}+1}{\xi^{ox}}\right)}^{2}\right]$$

where $\dot{\varepsilon_{th}}$ and $\dot{\varepsilon_{mech}}$ are the thermal strain rate and the strain rate, respectively, and $\dot{\varepsilon_{th}}/\dot{\varepsilon_{mech}}$ becomes 0 in the case of the isothermal LCF test. In addition, $\xi^{ox}$ is the relative amount of oxidation damage associated with the phase. Therefore, the growth of the oxide penetrating the material can be predicted using Equation (11).

Sehitoglu et al. proposed oxidation damage lifetime model in Equation (17) by reflecting the phenomenon where the oxide produced in a high-temperature environment penetrates the material under a load.

(17) $$\frac{1}{N_{env}}={\left[\frac{h_{cr}\delta_{0}}{B\Phi^{ox}(K_{peff}^{ox}+K_{peff}^{\gamma^{\prime }})}\right]}^{-\frac{1}{\beta }}\times \frac{2{\left(\Delta \varepsilon_{mech}\right)}^{\left(2/\beta \right)+1}}{\varepsilon_{mech}^{1-\alpha /\beta }}$$

Here, $N_{env}$ is the oxidation damage lifetime, and $h_{cr}$ is the critical oxidation layer thickness. Meanwhile, Sehitoglu et al. suggested oxidation damage parameters reflecting the $\gamma^{\prime }$ depleted layer inside the material with insufficient chromium, as summarized in Table 1. Before the sensitization temperature range, a $\gamma^{\prime }$ depleted layer lacking chromium is present between the material and Cr_23_C_6_ due to the effect of Cr_23_C_6_ existing on the surface of austenitic stainless steel [1,42]. In addition, in the sensitization temperature range (400~850 °C), there is a $\gamma^{\prime }$ depleted layer lacking chromium around the Cr_23_C_6_ formed inhomogeneously along the grain boundary and in the grains. Therefore, in this study, the oxidation damage parameters in Table 1 were used in consideration of the $\gamma^{\prime }$ depleted layer of austenitic stainless steel.

The Neu-Sehitoglu model is limited in reflecting the phenomena that occur in cracks such as crack opening and crack-tip blunting, while the mechanism-based lifetime model is limited in reflecting oxidation damage. Therefore, a unified fatigue life model was proposed as shown in Equation (1) by combining the fracture mechanism-based lifetime estimation and the effectiveness of Neu-Sehitoglu oxidation damage.

## 3. Experiments

### 3.1. Materials and Specimen

**Figure 1:**
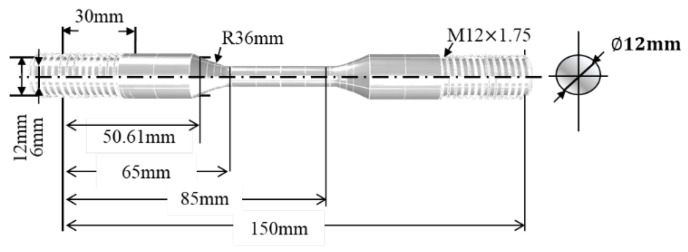
Specimen for the LCF test.

Table 2 shows the chemical composition of niobium-stabilized austenitic stainless steel which is similar to stainless steel 316 through the contents of large amounts of chromium, nickel, and molybdenum. Herein, 1.4~1.5 wt.% of niobium is also added to prevent depletion of chromium while improving heat resistance and corrosion resistance. Therefore, the specimen was cast in a Y-block form and air-cooled without special heat treatment. Figure 1 shows the specimen made of austenitic stainless steel for the LCF test, which was prepared in a cylindrical shape of Φ6 mm ×20 mm in gauge measurements according to ASTM E606 [43].

### 3.2. Experiment Procedure

**Figure 2:**
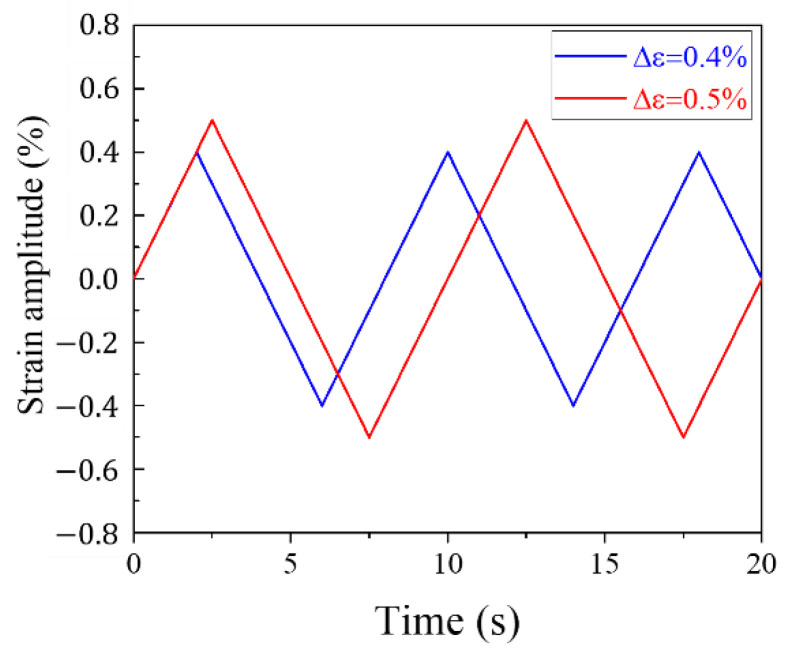
Strain amplitude for the LCF test.

In this study, the isothermal LCF test was performed in the strain-controlled method using a computer-controlled MTS axial servo-hydraulic tester (MTS Landmark 100 kN) with an axial load capacity of 100 kN. Figure 2 shows the strain amplitudes and waveforms. The strain amplitudes were set to 0.4% and 0.5% to verify the fatigue life prediction model while confirming the effect of the load at the strain rate of $0.002\;s^{-1}$. In addition, four temperature conditions of 200, 400, 600, and 800 °C were set to examine the oxidation behavior and fatigue life with increasing temperature, and the isothermal LCF test was performed by controlling the temperature with a 2-zone temperature control MTS furnace. To protect the fractured surface of the specimen, a fracture in the LCF test was defined as the point at which the maximum stress was reduced by 20%. The test was repeated at least 10 times under each temperature condition to ensure the reliability of the LCF test results, and the average LCF life was calculated. From the LCF tests, niobium-stabilized austenitic stainless steel shows cyclic hardening behavior at 400 °C and 600 °C, and as the cycle increases, the peak stress continuously increases and the specimen is eventually fractured. On the other hand, because of the cyclic softening of the material, the fatigue life determined before the 20% reduction of maximum stress at 200 °C and 800 °C is about 98% and 82% of the total cycles to fracture, respectively.

**Figure 3:**
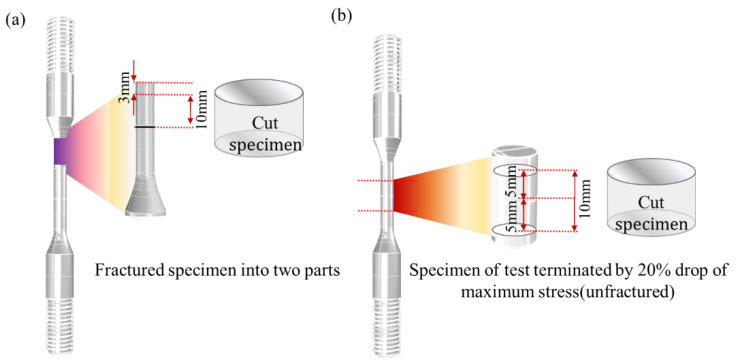
Schematic of cut specimen for microstructure analysis: (**a**) specimen separated into two parts; (**b**) unfractured specimen.

**Figure 4:**
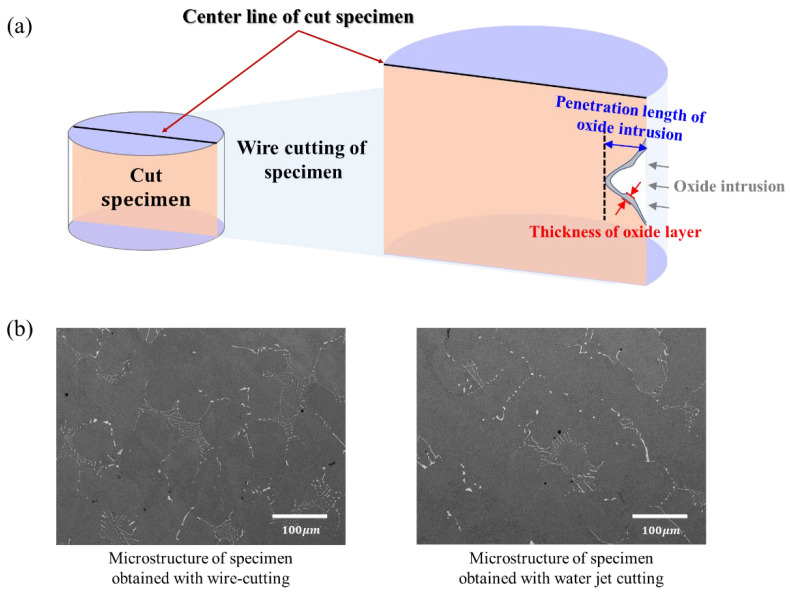
(**a**) Schematic of microstructural analysis: thickness of oxide layer and penetration length of oxide intrusion, and (**b**) results of microstructure analysis according to wire cutting and water jet cutting.

Finally, after the LCF test, microstructure observation and composition analysis were conducted to confirm the precipitation of carbide, the average thickness of the oxide layer in oxide intrusion, and the penetration length of oxide intrusion. Figure 3 shows the sampling method following the LCF test. If the specimen is fractured and separated into two parts (Figure 3a), cut it to a length of 10 mm at a distance of 3 mm from the fracture surface of the longer specimen part. On the other hand, the unfractured specimen (Figure 3b) is cut 5 mm up and down in the middle of the gauge length. Figure 4a shows the position of the microstructure analysis for the average thickness of the oxide layer, and the penetration length of oxide intrusion after wire cutting the specimen. In order to minimize heat transfer on the specimen, it was cut while soaked in oil, and there was no change in the microstructure because the diameter, 6 mm, of the specimen was small and the processing time was short. Although a very small amount of soot may accumulate on the surface as a result of the electrical discharge, it can be removed during the grinding and polishing process, and when compared to a specimen cut by a water jet, no difference in microstructure can be seen in the fractography analysis results, as shown in Figure 4b.

Microstructure analysis was performed using a scanning electron microscope (SEM, S8000G, TESCAN, Brno, Czech Republic), and the compositions of carbide precipitates and oxides were analyzed through Bruker’s energy-dispersive X-ray spectroscopy (EDS, QUANTAX EDS & EBSD, Bruker, Billerica, MA, USA) program and the X-ray diffraction patterns (XRD, Dmax2500/PC, Rigaku, Tokyo, Japan).

## 4. Results and Discussions

### 4.1. Microstructure Analysis

**Figure 5:**
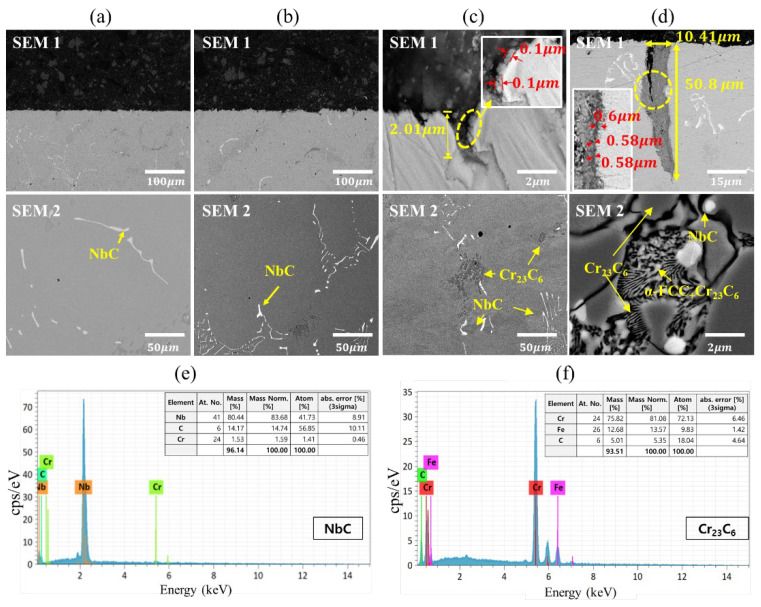
SEM-back scattered electron image of oxidation damage (SEM 1) and carbide precipitation (SEM 2) after the 0.4% strain amplitude LCF tests at (**a**) 200 °C; (**b**) 400 °C; (**c**) 600 °C; (**d**) 800 °C and EDS analysis of carbide precipitation of (**e**) NbC; (**f**) Cr_23_C_6_.

**Figure 6:**
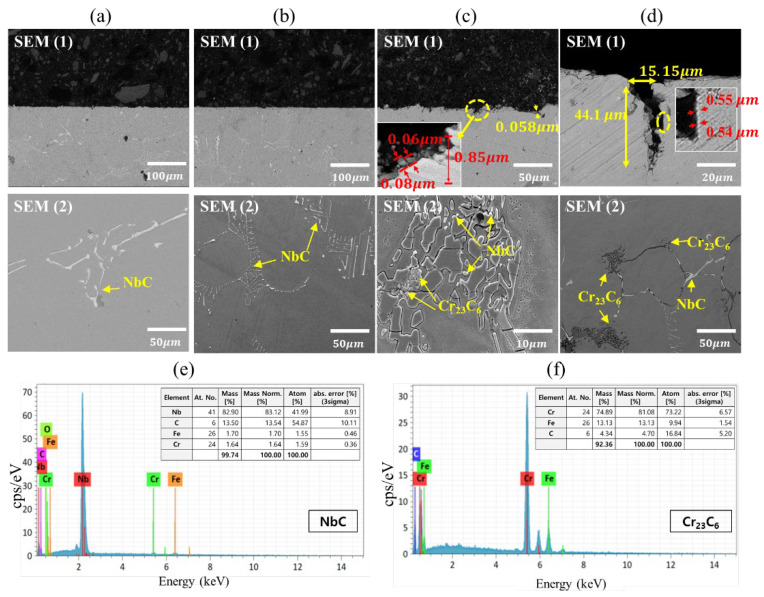
SEM-back scattered electron image of oxidation damage (SEM 1) and carbide precipitation (SEM 2) after the 0.5% strain amplitude LCF tests at (**a**) 200 °C; (**b**) 400 °C; (**c**) 600 °C; (**d**) 800 °C and EDS analysis of carbide precipitation of (**e**) NbC; (**f**) Cr_23_C_6_.

Figure 5 and Figure 6 show the microstructure and composition analysis results of the low-cycle fatigue test specimen at each temperature under strain amplitude conditions of 0.4% and 0.5%, respectively. In the case of 200 °C (Figure 5a and Figure 6a), no preparation of carbides occurred, such as M_23_C_6_, but a distribution of niobium carbide (NbC) was observed. This result is attributed to the low temperature and short time for Cr_23_C_6_ to precipitate, and no oxide could be observed as well. At 400 °C (Figure 5b and Figure 6b), while the temperature is in the sensitization temperature range of austenitic stainless steel (400–850 °C), no Cr_23_C_6_ was formed. Similar to 200 °C, the distribution of NbC was observed, which is believed to be a characteristic of the presence of niobium. In the sensitization temperature range (400–850 °C), the chemical affinity between carbon and chromium is relatively smaller at 400 °C than at other temperatures; however, because niobium has a stronger affinity with carbon than chromium, NbC appears to precipitate preferentially while suppressing the formation of Cr_23_C_6_. As a result, the chromium inside the material effectively served its role of inhibiting oxidation, and no oxide was observed at 400 °C.

Contrary to the experimental results at 200 °C and 400 °C, oxides were observed at 600 °C (Figure 5c and Figure 6c) in a thin, gray layer with a thickness of 0.1 μm and 0.06 μm on the surface of oxidation induced crack and penetration length of oxide intrusion was found to be 2.01 μm and 0.85 μm. In addition, Cr_23_C_6_ was also observed through EDS analysis [44,45]. When Nb is added to austenitic stainless, NbC precipitates preferentially as niobium, which has a stronger affinity with carbon than chromium, and bonds with carbon while suppressing the formation of Cr_23_C_6_ inside the material. For this reason, Cr_23_C_6_ seems to be generated at a temperature higher than the 400 °C and because the temperature range with the strongest binding force between carbon and chromium over the sensitization temperature range is 550–650 °C, it is believed that Cr_23_C_6_ was formed during the aging period while the fatigue test was being performed at 600 °C [46].

**Figure 7:**
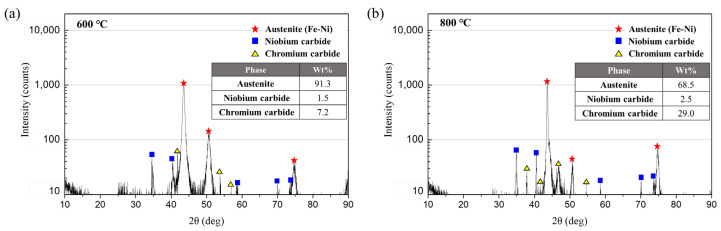
X-ray diffraction patterns and weight fraction of the phase of austenitic stainless steel with Nb; (**a**) LCF at 600 °C; (**b**) LCF at 800 °C.

Similar to 600 °C, the formation of Cr_23_C_6_ was observed at 800 °C (Figure 5d and Figure 6d), and the thickness of the oxide layer (0.6 μm and 0.54 μm) and the penetration length of oxide intrusion (50.8 μm and 44.1 μm) suggested that the oxidation reaction was more active compared to the experimental results at 600 °C. Particularly, the XRD analysis in Figure 7 indicated that more Cr_23_C_6_ was produced along with NbC at 800 °C than at 600 °C, which is attributed to the increasing chemical affinity of chromium with carbon in addition to that of niobium as the temperature rises. When an LCF is applied to the specimen at a high temperature, niobium reduces the depletion rate of chromium and increases the resistance to oxidation. However, as the temperature enhances, the chemical affinity of chromium and carbon of stainless steel increases, resulting in carbide precipitation such as Cr_23_C_6_, which causes oxidation damage along with the depletion of chromium.

As a result of the microstructure observation and composition analysis, the formation of carbides was found to increase at 600 °C or higher, and larger oxide layer thicknesses and cracks caused by oxidation were also observed, regardless of the effect of the strain amplitude. These results differ from typical austenitic stainless steel, which exhibits carbide formation and oxidation behavior at 400 °C, and it is believed that niobium with a stronger affinity with carbon than chromium reduced the amount of carbide formation and the extent of oxidation.

### 4.2. Oxidation Damage Analysis

Table 3 shows the average oxide layer thickness observed at the oxide intrusion in the LCF test and the oxide layer thickness predicted using the Neu-Sehitoglu oxidation model. The temperatures from 200 °C to 400 °C were too low for the specimens to form carbide precipitates, and the oxide layer thickness was too small to be observed. Under the two strain amplitude conditions, the average oxide layer thickness was 0.11 μm and 0.07 μm at 600 °C, respectively, and 0.6 μm and 0.54 μm at 800 °C, respectively. However, the oxide layer thickness predicted by the Neu-Sehitoglu model was 0 at 200 °C to 400 °C, 0.14 μm and 0.09 μm at 600 °C under the strain amplitudes of Δε=0.4% and Δε=0.5%, and 0.83 μm and 0.69 μm at 800 °C under the two strain amplitudes, respectively. As a result, both the average experimental value and predicted value tended to be larger at the strain amplitude of 0.4% than at the strain amplitude of 0.5%.

When the oxide is not damaged, the oxide growth of the austenitic stainless steel tends to increase in a parabolic shape, and the thickness of the oxide increases as shown in Equation (8). Also, an increase in strain amplitude leads to an enhancement of stress applied to the material, which speeds up the fatigue crack propagation rate. Therefore, it can be assumed that the oxide layer thickness is relatively thin because the specimen’s exposure time to the environment is short in the fatigue test at 0.5% strain amplitude compared to the 0.4% strain amplitude condition test, which can be inferred from Equation (14). Also, the thickness of oxide layer values predicted by adopting the Mar-M247 variables tends to be larger than the experimental values at 600 °C or higher. According to this result, when the oxide layer is not damaged, it may be seen that Mar-M247 has a larger $K_{p}^{ox}+K_{p}^{\gamma^{\prime }}$ value than niobium-stabilized austenitic stainless steel by Equation (8). As a result, using the Neu-Sehitoglu oxidation model, the oxide layer thickness at various temperatures and deformation amplitudes was predicted to have an average error of 14.7%.

Table 4 summarizes the penetration length of the oxide intrusion formed by the oxidation damage based on the average values from the test specimens and the predicted values from the Neu-Sehitoglu oxidation model. It was difficult to confirm the oxide intrusion from 200 °C to 400 °C at which no oxide was formed regardless of the amount of the strain amplitude. However, at 600 °C with the formation of oxide, 2.01 and 0.85 of the penetration lengths of oxide intrusion were observed at the strain amplitudes of 0.4% and 0.5%, respectively, and at 800 °C with an increased formation of oxide, the average penetration lengths of oxide intrusion were 50.8 μm and 44.1 μm. Conversely, the numerically predicted penetration length of oxide intrusion was 0 from 200 °C to 400 °C, 1.45 μm and 0.70 μm at 600 °C, and 35.6 μm and 31.6 μm at 800 °C.

According to Equation (11), as the thickness of the oxide layer decreases, oxide intrusion due to repeated rupture of the oxide layer increases. When LCF specimens under conditions of strain amplitude 0.4% and 0.5% are exposed to the oxidation environment for the same time, the thickness of the oxide layer under strain amplitude 0.5% condition is predicted to be smaller than the result of strain amplitude 0.4%, and the oxide growth rate is faster. However, since the aging time of the strain amplitude 0.4% specimens is relatively longer than those of the specimens at 0.5% condition, the penetration length of oxide intrusion was predicted as shown in Table 4.

Although the oxidation-induced damage was not observed up to 400 °C, which was similar in both experimental and predictions, the predicted values of the penetration length of oxide intrusion were smaller compared to the experimental values. When cracks grow due to oxidation by repeated formation and rupture of oxide, not only the value of $K_{p}^{ox}+K_{p}^{\gamma^{\prime }}$ but also the growth rate of the oxidation-induced crack may affect the error of a predicted value by Equation (11). If the crack growth rate of Mar-M247 is slower than that of niobium-stabilized austenitic stainless steel, the predicted value of the penetration length of oxide intrusion is smaller than the experimental value, which is consistent with the results of Table 4. Therefore, the error in the predicted value is caused by employing the Mar-M247 variables rather than the characterized variables for niobium-stabilized austenitic stainless steel. Also, the error is thought to be caused by the difference in the crack growth rate due to oxidation of the two materials and variable s($Q_{ox}$*,* $Q_{\gamma^{\prime }}$*,* $D_{ox}$*,* $D_{\gamma^{\prime }}$), which determine the value of $K_{p}^{ox}+K_{p}^{\gamma^{\prime }}$.

From the microstructure analysis results in Figure 5 and Figure 6, the average width of oxide intrusion for the 0.4% strain amplitude specimens was 0.5 μm and 10.41 μm at 600 °C and 800 °C, respectively, while that of the 0.5% strain amplitude specimens was 5.84 μm and 15.15 μm, indicating that the width of oxide intrusion was larger at the strain amplitude of 0.5% than at 0.4%. This appears to be the result of the mechanical load from the greater extent of the periodic deformation in the LCF test, causing wider oxide intrusion. Therefore, the width of oxide intrusion increases as the surface area of the material subject to oxidation increases with the formation of cracks. However, the larger the strain amplitude, the smaller the average oxide intrusion observed in the fatigue test. Similar to the reason for the difference in oxide layer thickness growth, the penetration length of oxide intrusion was relatively smaller at the strain amplitude of 0.5% than at 0.4% because an increase in strain amplitude under the same temperature condition accelerates the propagation of the fatigue crack, which fractures the specimen rapidly while limiting the amount of time available for the penetration of the oxide.

As a result, the penetration length of oxide intrusion at various temperatures and strain amplitudes was predicted with an average error of 13.0% using the Neu-Sehitoglu oxidation model.

### 4.3. Fatigue Life Prediction

In this study, the LCF life was predicted by proposing a unified fatigue life model coupled with the fracture mechanism-based lifetime model and the Neu-Sehitoglu oxidation damage model to reflect the damaging effects of fatigue and oxidation found in high-temperature LCF, then the accuracy of the prediction model was verified by comparing the numerically predicted fatigue life to the experimental result.

**Figure 8:**
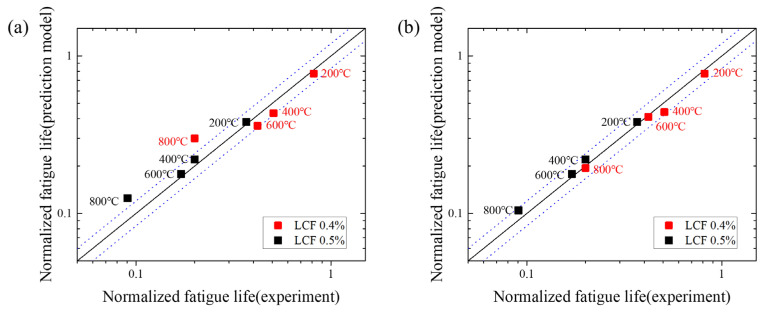
Experimental fatigue cycles to failure and lifetime model estimation for the LCF; (**a**) mechanism-based lifetime model only; (**b**) prediction model considering the oxidation damage.

The predicted and experimental values of the LCF test were normalized and compared to demonstrate the accuracy of the prediction model, as shown in Figure 8. The normalized fatigue lives are calculated by dividing the predicted and the representative experimental fatigue lives into the maximum fatigue life obtained in the entire LCF tests. When the oxidation damage was observed based on the microstructure analysis, it was confirmed that the smaller the strain amplitude under the same temperature condition, the thicker the oxide layer and the greater the penetration length of oxide intrusion. However, in the case of fatigue life, the larger the strain amplitude at the same temperature, the shorter the fatigue life. Although the thickness of the oxide layer and penetration length of oxide intrusion observed on the specimens were greater in the 0.4% strain amplitude test than in the 0.5% strain amplitude test, it is believed that the initiation and propagation of cracks were more rapid as the strain amplitude increased under the same temperature condition, resulting in shorter fatigue life. Also, as temperatures rise, a fatigue life decreases, and this is thought to be caused by accelerating the crack propagation as not only damage caused by fatigue but also damage caused by oxidation is increased.

Figure 8a shows the graphs comparing the predicted values using only the fracture mechanism-based lifetime model to the experimental results. The ideal prediction model with predicted values perfectly identical to the experimental values and the scatter band corresponding to the factor of 2 are indicated by solid and dotted lines, respectively. As a result of the LCF life prediction, the fatigue life was predicted within a factor of ±2 at all temperatures except for 800 °C, regardless of the strain amplitude.

While the fatigue tests at 400 °C and 600 °C were in the sensitization temperature range of austenitic stainless steel, the predicted values differed from the experimental results within a factor of ±2. As observed in the microstructure analysis result of the specimens tested at 400 °C, niobium played the role of suppressing the formation of Cr_23_C_6_ and oxide as much as possible, and oxidation damage was therefore minimal to observe. In addition, in the test conducted at 600 °C, the thickness of the oxide layer, penetration length of oxide intrusion, and width of damage by oxide formation are relatively small compared to the results at 800 °C because the amount of Cr_23_C_6_ precipitates was relatively small. This implies that, unlike at 800 °C, the damaging effect of oxidation in determining the fatigue life was insignificant, and it was possible to predict the fatigue life within a factor of ±2 only using the mechanism-based lifetime model.

**Figure 9:**
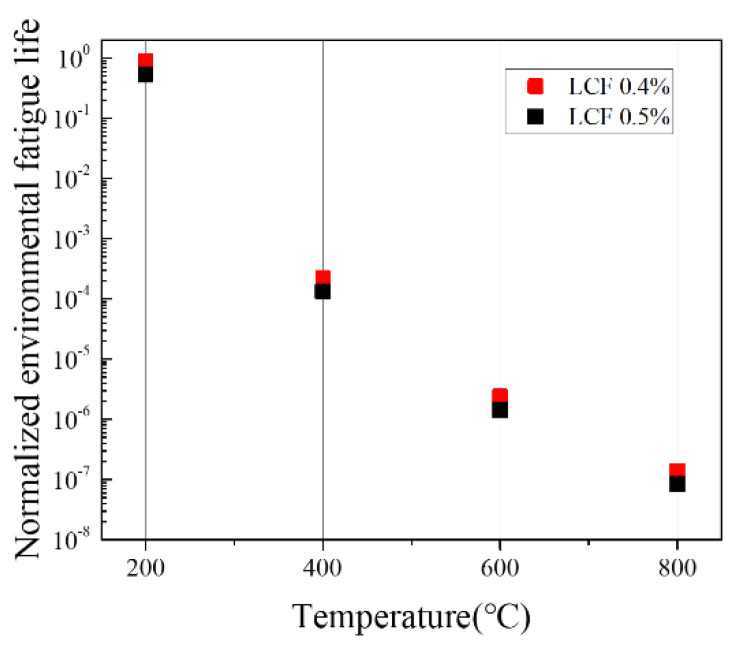
Normalized environmental fatigue life results calculated with Neu-Sehitoglu oxidation damage model.

However, as the temperature increased in the LCF test, the damage by oxidation increased in the austenitic stainless steel containing niobium, and as explained in the previous section, the formation of Cr_23_C_6_ and oxides increased at 800 °C, resulting in a greater the thickness of oxide layer and penetration length of oxide intrusion than at other lower temperatures. The prediction accuracy appears to be lower because the effect of oxidation damage is not properly reflected in the fatigue life prediction model, and Figure 8a confirms that the predicted fatigue life at 800 °C was higher than the experimental result. Figure 9 shows the normalized fatigue life calculated in the Neu-Sehitoglu model, which indicates that the oxidation-induced environmental fatigue life decreased with increasing temperature, and the effect of oxidation was maximized at 800 °C as the fatigue life rapidly decreased. From Equation (17), the variables except for effective hyperbolic oxidation coefficient, $K_{peff}^{ox}$*,* and effective hyperbolic depleted layer coefficient, $K_{peff}^{\gamma^{\prime }}$, are the same at all temperatures under the same strain amplitude condition. Accordingly, it is thought that the environmental fatigue life is greatly affected by the temperature-dependent exponential function of the Neu-Sehitoglu model. Meanwhile, the intensive formation of chromium carbides at 400 °C has not yet been identified, but the fatigue life at 400 °C is reduced by 1/4005 of the fatigue life at 200 °C in Figure 9.

Therefore, a fatigue life prediction model that can reflect the damage caused by oxidation should be applied to predict the LCF life at high temperatures; Figure 8b shows the predicted fatigue life results from the unified model coupled with the fracture mechanism-based lifetime model and the Neu-Sehitoglu model. As shown in the graph, the fatigue life can be predicted within the error of a ±2 factor in the temperature range of 200 °C to 800°C under the strain amplitude conditions of both 0.4% and 0.5%. This is believed to be the result of adequately reflecting the effect of fatigue and the characteristics of oxidation damage.

### 4.4. Sensitivity Analysis of Oxidation Damage Parameters

In this study, the Neu-Sehitoglu oxidation damage model adopted to consider the effect of oxidation on fatigue life prediction is proposed to calculate the isothermal LCF life as well as the fatigue life under the TMF load conditions that control the temperature and strain rate over time.

To develop an accurate model, experiments are essential to identify the parameters that can reflect the effect of temperature, strain rate and oxidation damage for niobium-stabilized austenitic stainless steel. Although it is unnecessary to consider in detail the effects of thermal changes or the phase between the temperature and strain in an isothermal LCF test, because it is difficult to attain parameters specific to niobium-stabilized austenitic stainless steel by performing experiments reflecting all possible changes in load and temperature conditions, the oxidation damage variables presented by Sehitoglu for the Mar-M247 material in Table 1 were used in this study for predicting fatigue life.

However, the use of the parameters for Mar-M247 in the Neu-Sehitoglu oxidation damage model can lead to errors in predicting the exact fatigue life because the parameters are not specific to niobium-stabilized austenitic stainless steel, and the sensitivity analysis proposed by Amaro et al. was conducted to investigate the effect of the changes in oxidation damage parameters on the final fatigue life [47]. To this end, a specific parameter was varied by a certain amount while maintaining the remaining input values among the parameters for Mar-M247, and each input value used for sensitivity analysis was increased by 0.5% over the minimum range of +/−40% to observe the changes in fatigue life. In this study, five parameters considered to be important for the oxidation characteristics of the material in the Neu-Sehitoglu oxidation damage model were selected and used as the input values for the sensitivity analysis. Amaro et al. also conducted a sensitivity analysis on all parameters in the Neu-Sehitoglu model, and among them, $Q_{ox}$, $Q_{\gamma^{\prime }}$, α, and β were the variables that had the highest effect on predicting fatigue life due to oxidation damage. In this study, variable sensitivity analysis was performed in the same way as Amaro et al.’s method to confirm the suitability of using oxidation-related variables of Mar-M247 to predict the fatigue life of niobium-stabilized austenitic stainless steel. However, since LCF tests were performed at constant strain rate and phase conditions, the effect on strain rate sensitivity, α, was excluded. Therefore, in order to consider material characteristics affecting the oxide formation and the oxide-induced crack growth, activation energies ($Q_{ox}$ and $Q_{\gamma^{\prime }}$), diffusion coefficients ($D_{ox}$ and $D_{\gamma^{\prime }}$) and oxidation-induced rate exponent (β) were employed for sensitivity analysis of damage parameters as follows:

- Oxidation-induced rate exponent, β
- Activation energy for oxidation, $Q_{ox}$
- Activation energy for $\gamma^{\prime }$ depletion, $Q_{\gamma^{\prime }}$
- Diffusion coefficient for oxidation, $D_{ox}$
- Diffusion coefficient for $\gamma^{\prime }$ depletion, $D_{\gamma^{\prime }}$

**Figure 10:**
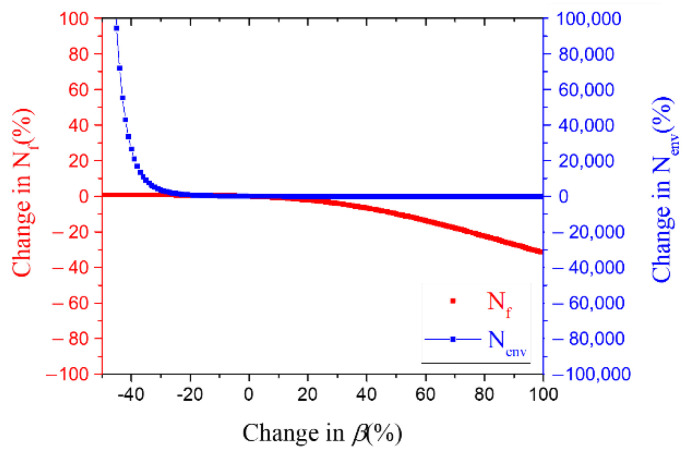
Fatigue life according to oxidation-induced rate exponent, *β* changes based on sensitivity analysis.

Figure 10 shows the oxidation fatigue life and the final fatigue life calculated in the Neu-Sehitoglu oxidation damage model when the oxidation-induced rate exponent, β, was varied from −40% to 100% of the normal value. In this case, when β was reduced by 20% or more, the fatigue life by oxidation drastically increased, but there was little change in the overall fatigue life. When β was increased by 10% or more, the fatigue life by oxidation as well as the overall fatigue life was reduced.

**Figure 11:**
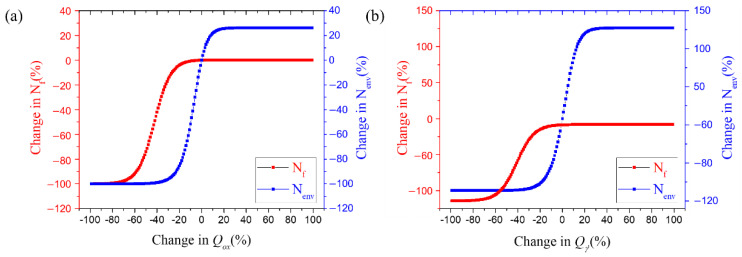
Fatigue life according to activation energy variable changes based on sensitivity analysis; (**a**) $Q_{ox}$, (**b**) $Q_{\gamma^{\prime }}$.

**Figure 12:**
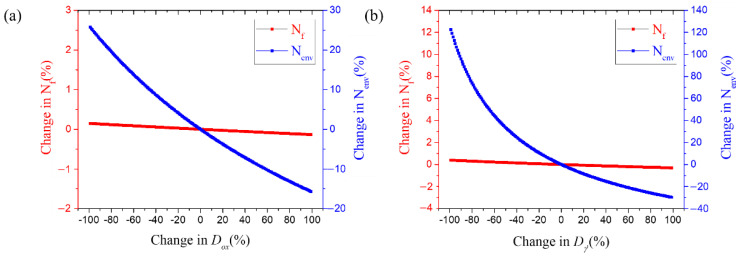
Fatigue life according to diffusion coefficient variable changes based on sensitivity analysis; (**a**) $D_{ox}$, (**b**) $D_{\gamma^{\prime }}$.

As shown in Figure 11, the fatigue life and the final fatigue life depend on the rate of changes in the activation energy for material oxidation and $\gamma^{\prime }$ depletion exhibited similar change patterns. When $Q_{ox}$ and $Q_{\gamma^{\prime }}$ were varied by +/−25%, the fatigue life by oxidation also increased and decreased in the same manner whereas it converges to a constant value with changes of +/−25% or more. The final fatigue life was found to decrease with the fatigue life by oxidation as the activation energy decreased, but an increase in the parameter value did not affect the final fatigue life. Conversely, the changes in the diffusion coefficients, $D_{ox}$ and $D_{\gamma^{\prime }}$, had an insignificant effect on the final fatigue life compared to the other parameters, as shown in Figure 12.

Abdullahi O. Abu performed sensitivity analysis by including the strain sensitivity constant, b, and the shape factor, $\varsigma_{ox},$ as a measure of the relative amount of damage with the different phases, which were not included in the sensitivity analysis of this study given the constant strain rate and phase (i.e., no temperature change over time) [48]. As a result, the oxidation-induced rate exponent, β, was found to have the greatest effect on lifetime prediction, followed by the activation energy for $\gamma^{\prime }$ depletion, $Q_{\gamma^{\prime }}$, the activation energy for oxidation, $Q_{ox}$, and the two diffusion coefficients, $D_{ox}$ and $D_{\gamma^{\prime }}$.

As can be seen from Equation (11), the oxidation-induced rate exponent, β, is a variable that affects the rate of oxide growth, and as the value increases, oxide-induced cracks also grow rapidly and the fatigue life due to oxidation decreases. These characteristics seem to have been confirmed through Figure 10. During the LCF test run time, *t*, of niobium-stabilized austenitic stainless steel, the predicted penetration length of oxide was within 30% of the error. In order to obtain the maximum rate of change for β, the rate of change equation could be expressed as $\left|\frac{t^{1.5}-t^{\beta }}{t^{1.5}}\right|\leq 0.3$, under the condition that all variables related to crack growth except β are the same. Based on the rate of change equation, the β value of the niobium-stabilized austenitic stainless steel for the time (3500 to 15,500 s) during the LCF test is up to 3% greater than the value of Mar-M247, and this change reduces 21% of the environmental fatigue life, $N_{env}$. However, from Equation (7) and Equation (17) in LCF, the $N_{env}$ is bigger than the $N_{fatigue}$ predicted by the fracture mechanism-based lifetime model, so it has a minor effect on the prediction of the final fatigue life, $N_{f}$, which can be seen in Figure 10.

In addition, the growth of cracks is correlated with the effective hyperbolic coefficient, $K_{peff}^{ox}$ and $K_{peff}^{\gamma^{\prime }}$. The activation energy in the material and the depleted layer plays an important role in generating oxidation damage, and the larger the value, the more energy is required for oxidation, so the resistance to fatigue is increased However, as the diffusion coefficient increases, the repeated formation and breakage of oxide increases, as does the growth rate of the oxide-induced crack.

Because of these characteristics, the values of the activation energy and diffusion coefficient affect oxidation damage, which can be shown in Figure 11 and Figure 12. However, typical austenitic stainless steel, such as stainless steel 316 and 304, is known to have an activation energy greater than that of Mar-M247 [17,49], and the addition of niobium is thought to have increased activation energy. Therefore, if the activation energy increases, $N_{env}$ can be enhanced by up to 20%, but the change in $N_{f}$ is expected to be very small through Figure 11.

In conclusion, by performing variable sensitivity analysis, the effect of changes in Mar-M247 oxidation-related variables on the fatigue life of niobium-stabilized austenitic stainless steel was investigated, and the suitability of using the Mar-M247 variable for fatigue life prediction was confirmed. Since Mar-M247 and niobium-stabilized austenitic stainless steel are different materials, errors can occur by up to 30% in predicting the amount of oxidation damage. However, the tendency to increase oxidation damage due to temperature rise could be properly predicted, and the fatigue life could be predicted well within a factor of 2 at temperatures ranging from 200 to 800 °C by using the unified fatigue life prediction model in conjunction with the fracture mechanism-based fatigue life and environmental fatigue life from the Neu-Sehitoglu model.

## 5. Conclusions

An isothermal low-cycle fatigue test was performed on austenitic stainless steel containing niobium at various temperatures and strain amplitudes. Based on the results of the microstructure observation and composition analysis, the formation of oxides with increasing temperature was confirmed, and the suitability of the Neu-Sehitoglu oxidation damage model for predicting the thickness of the oxide layer and the penetration length of oxide intrusion was examined. In addition, a unified low-cycle fatigue life model was proposed to couple the fracture mechanism-based lifetime and the Neu-Sehitoglu oxidation damage, and its validity was investigated. The main conclusions are as follows.

(1) As a result of the microstructure observation and composition analysis, the thickness of the oxide layer and penetration length of oxide intrusion were observed through the formation of oxides at 600 °C and higher, regardless of the effect of the strain amplitude. This observation differs from the behavior of typical austenitic stainless steel such as stainless steel 304 or 316, which develops oxides at 400 °C or higher, and it appears to result from the suppression of the formation of Cr_23_C_6_ that is associated with the oxidation of the material by niobium addition.
(2) In order to suppress sensitization, niobium is added to the austenitic stainless steel, and because niobium has a stronger affinity with carbon than chromium, NbC appears to precipitate preferentially while suppressing the formation of Cr_23_C_6_. As a result, the chromium inside the material effectively served its role of inhibiting oxidation, and accordingly, no oxide was observed at 200~400 °C. However, at 600 °C or higher, the affinity between carbon and chromium becomes stronger, so Cr_23_C_6_ was formed during the aging time and oxidation damage associated with deficiency of chromium.
(3) The comparison of the predicted and experimental values of oxidation damage in the low-cycle fatigue test indicated that the average thickness of the oxide layer and the penetration length of oxide intrusion were evaluated with an average error range of 14.7% and 13.0%, respectively. The thickness of the stratified oxide layer in oxide intrusion of austenitic stainless steel decreases as the strain amplitude increases at the constant strain rate. In the Neu-Sehitoglu model, the average oxide layer thickness was the same as the experimental tendency. In addition, the Neu-Sehitoglu oxidation damage model can take into account both average oxide thickness and aging time in calculating the rate of oxide growth, so the penetration length of oxide intrusion seems to have been well predicted. Based on the above results, it is believed that the oxidation characteristics of austenitic stainless steel containing niobium, which forms an oxide at 600 °C and higher, can be adequately predicted in the Neu-Sehitoglu model.
(4) The predicted low-cycle fatigue lives with and without using the Neu-Sehitoglu oxidation damage model were compared. As a result, it was confirmed that fatigue life could be predicted within a factor of ±2 at all temperatures except for 800 °C, regardless of the strain amplitude. Usually, in a certain temperature region where fatigue life is less affected by oxidation damage, the estimation of a lifetime through the fracture mechanism with crack-tip displacement theory can be employed to predict LCF. However, austenitic stainless steel containing niobium begins to be damaged by oxidation at 600 °C, and higher, a large amount of Cr_23_C_6_ is precipitated and the damage caused by oxidation increases rapidly. In order to predict the fatigue life of austenitic stainless steel much more accurately at overall temperatures, thus combining oxidation damage with fracture mechanism-based lifetime was found to be essential, and it was possible by using this unified low-cycle fatigue life prediction model.
(5) Sensitivity analysis was performed on five parameters applied in the Neu-Sehitoglu oxidation damage model, which confirmed that the oxidation-induced rate exponent, β, had the greatest effect in predicting the low-cycle fatigue life at a constant strain rate and under the dT/dt=0 condition with no temperature change. Furthermore, because the activation energy for oxidation of austenitic stainless steel is larger than the value of Mar-M247, the parameter is believed to have an insignificant effect on the prediction of fatigue life.
(6) A unified fatigue life model was proposed to properly reflect the material damage and fatigue failure in a high-temperature, and repeated-load environment. It is expected that this model will allow a more accurate prediction of the fatigue life of austenitic stainless steel components containing niobium, such as automotive engines, internal combustion engines of transportation vehicles, and gas turbines.

## Figures and Tables

**Table 1:** Oxidation damage variables presented by Sehitoglu for Mar-M247, adapted with permission from Ref. [16]. Copyright 1990, American Society of Mechanical Engineers.

Parameter		Value
B	Time to reach critical oxide layer thickness	6.93 × 10^−3^ (s^−0.5^)
$\delta_{0}$	Measurement of oxide ductility	1.12 × 10^−10^ (μm·s^−0.75^)
α	Strain rate sensitivity	0.75
β	Oxidation-induced rate exponent	1.5
$h_{cr}$	Critical total oxide growth	461.4 (μm)
$\varsigma_{ox}$	Shape factor as a measure of the relative amount of damage with the different phase	0.44
$D_{ox}$	Diffusion coefficient for oxidation	1.54 × 10^4^ (μm^2^/s)
$D_{\gamma^{\prime }}$	Diffusion coefficient for $\gamma^{\prime \prime }$ depletion	8.57 × 10^3^ (μm^2^/s)
$Q_{ox}$	Activation energy for oxidation	175.9 (kJ/mol)
$Q_{\gamma^{\prime }}$	Activation energy for $\gamma^{\prime \prime }$ deplation	163.3 (kJ/mol)

**Table 2:** Chemical composition of austenite stainless steel.

**Composition** **[wt.%]**	**C**	**Si**	**Mn**	**P**	**S**	**Cr**	**Ni**	**Mo**	**Nb**	**Fe**
	0.3	2.5	2.0	0.04	0.04	18–23	8.8	0.16	1.4–1.5	Bal.

**Table 3:** Results of average thickness of oxide layer obtained from experiments and predictions.

Strain Amplitude	Temp (°C)	Experiment (μm)	Prediction (μm)	Error (%)
Δε=0.4%	200	0	0	0
	400	0	0	0
	600	0.11	0.14	27.3
	800	0.62	0.83	33.9
Δε=0.5%	200	0	0	0
	400	0	0	0
	600	0.07	0.09	28.6
	800	0.54	0.69	27.8
Average error(%)				14.7

**Table 4:** Results of penetration length of oxide intrusion obtained from experiments and predictions.

Strain Amplitude	Temp (°C)	Experiment (μm)	Prediction (μm)	Error (%)
Δε=0.4%	200	0	0	0
	400	0	0	0
	600	2.01	1.45	27.9
	800	50.8	35.6	29.9
Δε=0.5%	200	0	0	0
	400	0	0	0
	600	0.85	0.70	17.6
	800	44.1	31.6	28.3
Average error(%)				13.0

## Data Availability

Not applicable.
